# Development and validation of a portable articulated dynamometry system to assess knee extensor muscle strength

**DOI:** 10.1038/s41598-023-39062-0

**Published:** 2023-07-23

**Authors:** Youho Myong, Sungwoo Park, Minwoo Cho, Seung Yeon Cho, Woo Hyung Lee, Byung-Mo Oh, Sungwan Kim

**Affiliations:** 1 Department of Biomedical Engineering, Seoul National University College of Medicine, 101 Daehak-ro, Jongno gu, Seoul, 03080 Republic of Korea; 2 Department of Rehabilitation Medicine, Seoul National University Hospital, Seoul National University College of Medicine, 101 Daehak-ro, Jongno gu, Seoul, 03080 Republic of Korea; 3 Graduate School, Interdisciplinary Program in Bioengineering, Seoul National University, Seoul, 08826 Republic of Korea; 4 Institute of Innovative Medical Technology, Seoul National University Hospital Biomedical Research Institute, Jongno gu, Seoul, 03122 Republic of Korea; 5 Department of Transdisciplinary Medicine, Seoul National University Hospital, 101 Daehak-ro, Jongno gu, Seoul, 03080 Republic of Korea; 6 Department of Medicine, College of Medicine, Seoul National University, Seoul, 03080 Republic of Korea; 7 Department of Rehabilitation Medicine, National Traffic Injury Rehabilitation Hospital, Yangpyeong, Gyeonggi 12564 Republic of Korea; 8 Institute of Bioengineering, Seoul National University, 101 Daehak-ro, Jongno gu, Seoul, 03080 Republic of Korea

**Keywords:** Diagnosis, Quality of life, Musculoskeletal system

## Abstract

Muscle strength assessment is important in predicting clinical and functional outcomes in many disorders. Manual muscle testing, although commonly used, offers suboptimal accuracy and reliability. Isokinetic dynamometers (IKDs) have excellent accuracy and reliability; but are bulky and expensive, offering limited accessibility. This study aimed to design a portable dynamometer that is accessible, accurate and reliable, and to validate the device in a general population. The portable articulated dynamometry system (PADS) is a portable device with an embedded high-precision load cell, designed to measure muscle strength with optimal accuracy. Seventy-two participants underwent maximal isometric and isokinetic knee extensor torque measurement with the PADS and IKD, respectively. The PADS results were cross-validated against IKD results using change in mean (CIM). Interrater and intra-rater reliabilities were assessed using intraclass correlation coefficients, standard error of measurement, and minimal detectable change. The PADS maximal knee extensor strength results were not significantly different from those by IKD (CIM: − 2.13 Nm; 95% CI − 4.74, 0.49 Nm). The PADS showed interrater reliability (Pearson’s r: 0.958; ICC: 0.979; SEM: 5.51%) and excellent intra-rater reliability (Pearson’s r: 0.912; ICC: 0.954; SEM: 8.38%). The proposed PADS may be an effective alternative to IKD, offering good accuracy, reliability, and potentially better accessibility.

## Introduction

Muscle strength has been reported to be an independent predictor of clinical and functional outcomes in many disorders^1^. Even in healthy populations, lower muscle strength was a risk factor of all-cause mortality^1,2^. In a geriatric population with advanced cancer, better muscle strength was reported to be associated with prolonged survival^3^, better functional outcomes^4^, and improved quality of life^5^. Declining muscle strength was shown to be a risk factor for declining cognition and Alzheimer disease^6^, and even in early adulthood, muscle strength seems to affect the long-term risk of stroke^7^.

For muscle assessment, the quadriceps muscles have been identified as an effective muscle group to predict declining muscle mass and strength in various chronic disorders^3,8–10^. In their recently revised guideline in assessing sarcopenia severity, the European Working Group on Sarcopenia in Older People (EWGSOP2) included many gait performance scales (gait speed test, short physical performance battery, timed up and go test, and 400-m walk test), which are highly affected by the quadriceps strength^11^. In emergency departments and neurocritical care settings, acute deterioration of medical and/or neurological outcomes is often accompanied by a sudden declination in quadriceps strength^12,13^. In clinical settings, quadriceps strength assessment is most often performed using manual muscle testing (MMT)^14^. MMT is desirable because it is quick, intuitive, and easy to perform; however, critical drawbacks are that it is qualitative and examiner dependent^15^. MMT can only be properly executed if the examiner is stronger than the examinee; otherwise, the examiner cannot reliably discern partially impaired from normal muscle strength^16^. Further, because MMT is only semi-quantitative, it is impossible to reliably track the temporal change in muscle strength. Quantitative muscle testing using hand-held dynamometers (HHDs) allows precise monitoring of a participant’s muscle strength^14^; however, its reliability is also limited by the examiner strength. When evaluating a powerful muscle group such as the quadriceps, the accuracy and reliability of HHDs are often suboptimal^17^.

Commercially available isokinetic dynamometers (IKD) such as the Biodex System 4 Pro or BTE PrimusRS are accurate and reliable, and are often used as the “gold standard” for muscle strength testing^14,18,19^. While IKDs are accurate, they are substantially larger in size than HHDs and therefore not portable. IKDs are also significantly more expensive to use than HHD or MMT. Due to their size and cost, IKD often only offers limited accessibility and are not suitable for routine clinical monitoring of muscle strength^18^.

Several muscle strength assessment devices have been proposed to overcome the limited accuracy and reliability of MMT and HHDs, as well as the limited accessibility of IKDs. Sung et al.^18^ proposed an anchoring frame that aides the positioning of HHDs for patients in the supine position, while Hogrel et al*.*^14^ developed a portable dynamometer with load cell and in-house user interface to measure the knee extensor torque of participants in the sitting position. Hirano et al.^20^ used a belt-stabilized HHD to measure the sitting quadriceps strength with maximal portability, and by sacrificing some portability, Padulo et al*.*^21^ designed an isometric bench on which participants could sit to assess knee extension power. While these inventions have their respective strengths, they also have drawbacks. Although the HHD anchoring frame can cater to patients in supine position, it is too bulky to be considered portable. Other designs are both portable and accurate, but still require fixing to a bedframe and/or bench; further, they require participants to be able to sit, thus rendering patients with severe muscle weakness unable to be assessed.

The current article proposes a portable articulated dynamometry system (PADS) for routine monitoring of knee extensor torque. The PADS is a foldable frame embedded with a high precision loadcell. The articulated frame and light aluminum body allows maximal portability, and the system can be fastened to the participant’s limb, removing the need for ground fixation and allowing measurement in the supine position. The aim of this study was to design a portable articulated dynamometry device, evaluate the accuracy and reliability of the system via a cross-validation against the Biodex System 4 Pro, and report the results of a user experience survey.

## Results

Seventy-two healthy adults (50.0% female; mean age: 29.32 ± 4.57 years) participated in this study; 40 participants (55.6%) were assessed for intra-rater reliability. As all participants were measured twice on each knee to assess interrater reliability, and a subset (n = 40) went through two discrete assessments for intra-rater reliability, a total of 448 observations were gathered for each dynamometer. The age, sex, height, weight, and maximal grip strength of the study participants are presented in Supplementary Table 1.

**Figure 1:**
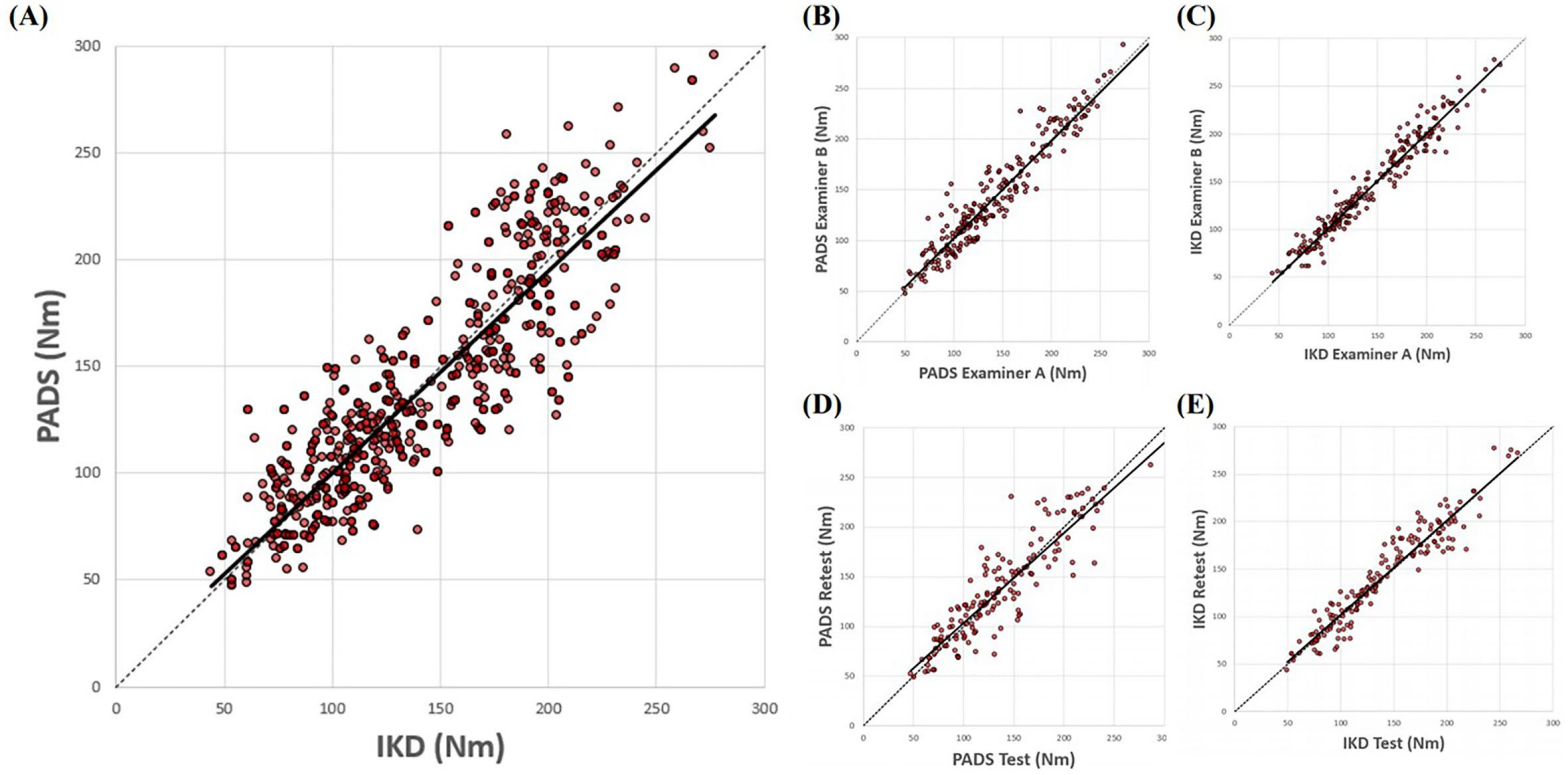
Linear regression analyses of the agreement between the PADS and IKD (**A**), interrater reliability of the PADS (**B**) and IKD (**C**), and intra-rater reliability of the PADS (**D**) and IKD (**E**). Dotted line is the identity line, while solid line represents the linear regression line.

**Figure 2:**
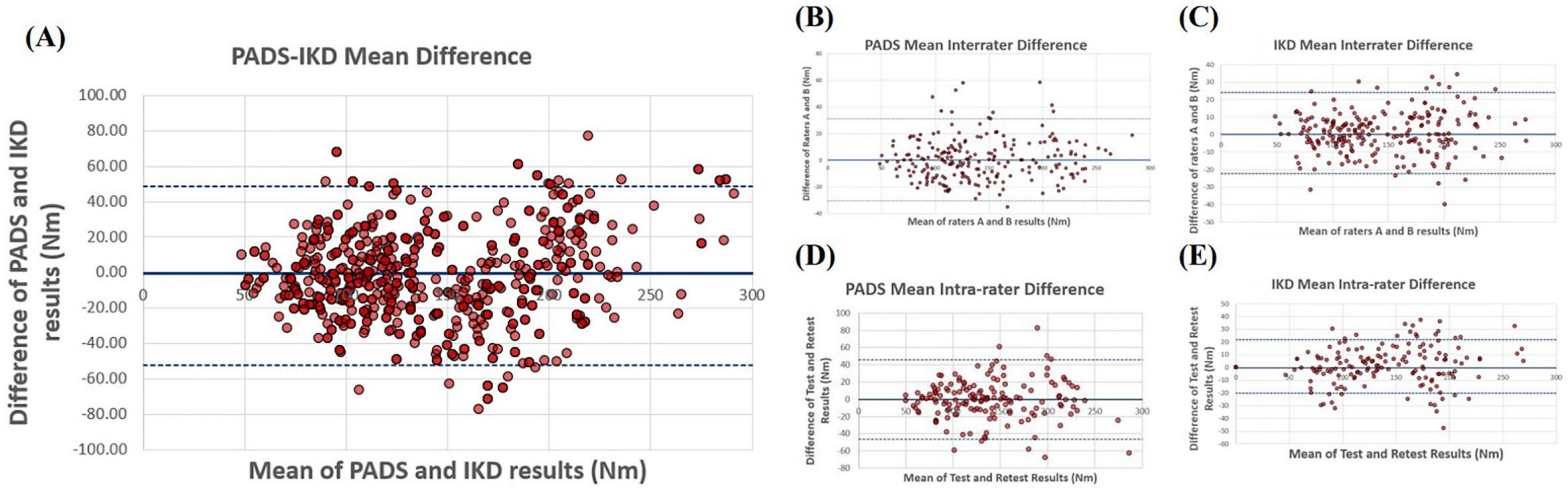
Bland–Altman charts of the agreement between the PADS and IKD (**A**), interrater reliability of the PADS (**B**) and IKD (**C**), and intra-rater reliability of the PADS (**D**) and IKD (**E**). Dotted lines are the limits of agreement, and solid line indicates the mean of difference.

Cross-validation results of the PADS versus IKD are summarized in Tables 1, 2 and 3 and Figs. 1 and 2. After 448 observations, the maximal knee extensor torque measured with the PADS was not significantly different from that measured with IKD (mean of the differences: − 2.13 Nm; 95% CI − 4.74, 0.49 Nm). Data measured with the PADS also demonstrated a strong correlation with data measured using the IKD (Pearson’s r: 0.880; *p* < 0.0001). The ICC between the PADS and IKD was 0.935, which indicates excellent correlation, and the mean SEM was 13.96 Nm, or 9.75%. The minimal detectable change (MDC) was 38.70 Nm. In terms of interrater reliability, the PADS showed no significant interrater difference (mean of the differences: 0.26 Nm; 95% CI − 1.83, 2.36 Nm), nor did the IKD (mean of the differences: 0.90 Nm; 95% CI: − 0.66, 2.46 Nm). Both systems showed strong interrater correlation (Pearson’s r: PADS, 0.958; IKD, 0.973) and excellent relative reliability (ICC: PADS, 0.979; IKD, 0.986). The mean SEM was 7.94 Nm (5.51%) for the PADS and 6.06 Nm (4.20%) for IKD. The MDC was 22.01 Nm (15.25%) for the PADS and 16.80 Nm (11.64%) for the IKD.

**Table 1:** Agreement of measurements between the PADS and IKD (n = 72 participants with a total of 448 measurements).

PADS (Nm)	IKD (Nm)	CIM (Nm) [95% CI]	Pearson’s* r*	*p*-value	ICC_2, k_	SEM (Nm)	MDC (Nm)
143.03 ± 54.62	145.16 ± 52.12	− 2.13 [− 4.74, 0.49]	0.880	< 0.0001	0.935	13.96 (9.75%)	38.70 (26.66%)

CIM, change in mean; ICC, intraclass correlation coefficient; IKD, isokinetic dynamometer; PADS, portable articulated dynamometry system; SEM, standard error of measurement; MDC, minimal detectable change.

**Table 2:** Interrater reliability of the PADS and IKD (n = 72, total 448 measurements).

Device	Examiner A	Examiner B	CIM (Nm) [95% CI]	Pearson’s* r*	*p*-value	ICC_2, k_	SEM (Nm)	MDC (Nm)
PADS	144.35 ± 54.90	144.09 ± 54.80	0.26 [− 1.83, 2.36]	0.958	< 0.0001	0.979	7.94 (5.51%)	22.01 (15.25%)
IKD	144.36 ± 51.23	143.47 ± 50.24	0.90 [− 0.66, 2.46]	0.973	< 0.0001	0.986	6.06 (4.20%)	16.80 (11.64%)

CIM, change in mean; ICC, intraclass correlation coefficient; IKD, isokinetic dynamometer; PADS, portable articulated dynamometry system; SEM, standard error of measurement; MDC, minimal detectable change.

**Table 3:** Intra-rater reliability of the PADS and IKD (n = 40, total 160 measurements).

Device	Test (Nm)	Retest (Nm)	CIM (Nm) [95% CI]	Pearson’s* r*	*p*-value	ICC_3,k_	SEM (Nm)	MDC (Nm)
PADS	139.54 ± 54.53	139.82 ± 54.64	− 0.28 [− 1.83, 2.36]	0.912	< 0.0001	0.954	11.70 (8.38%)	32.43 (23.24%)
IKD	142.93 ± 51.67	141.22 ± 49.63	1.72 [− 0.71, 4.14]	0.955	< 0.0001	0.977	7.83 (5.48%)	21.70 (15.18%)

CIM, change in mean; ICC, intraclass correlation coefficient; IKD, isokinetic dynamometer; PADS, portable articulated dynamometry system; SEM, standard error of measurement; MDC, minimal detectable change.

For intra-rater reliability, both the PADS and IKD showed no significant systematic bias between two different test dates (mean of the differences = − 0.28 Nm and 1.72 Nm; 95% CI [− 1.83, 2.36] Nm, [− 0.71, 4.14] Nm, respectively). Both systems showed strong interrater correlation (Pearson’s r: PADS, 0.912; IKD, 0.955) and excellent relative reliability (ICC: PADS, 0.954; IKD, 0.977). The mean SEM was 11.70 Nm (8.38%) for the PADS and 7.83 Nm (5.48%) for IKD. The MDC was 32.43 Nm (23.24%) for the PADS and 21.70 Nm (15.18%) for the IKD.

Table 4 reports the UEQ-S feedback; 69 of 72 participants completed the survey, and results reported significantly more positive overall user experience (*p* = 0.002) for the PADS. Specifically, participants responded that the PADS was significantly easier (*p* < 0.001), more efficient (*p* = 0.018), clearer (*p* = 0.027), more inventive (*p* = 0.002), and more leading-edge (*p* < 0.001) than the IKD.

**Table 4:** UEQ-S results (n = 69).

	IKD	PADS	CIM [95% CI]	*p*-value
Supportive	1.26 ± 1.22	1.26 ± 1.34	0.00 [− 0.32, 0.32]	1.000
Easy	0.65 ± 1.77	1.71 ± 1.34	1.06 [0.62, 1.50]	< 0.001
Efficient	1.07 ± 1.38	1.54 ± 1.26	0.46 [0.08, 0.85]	0.018
Clear	1.30 ± 1.33	1.71 ± 1.21	0.41 [0.05, 0.76]	0.027
Exciting	1.24 ± 1.38	1.26 ± 1.35	0.03 [− 0.29, 0.35]	0.857
Interesting	1.25 ± 1.42	1.38 ± 1.33	0.13 [− 0.21, 0.47]	0.449
Inventive	0.87 ± 1.53	1.48 ± 1.32	0.61 [0.23, 0.99]	0.002
Leading edge	0.61 ± 1.37	1.32 ± 1.22	0.71 [0.36, 1.06]	< 0.001
Total score	8.01 ± 9.58	11.58 ± 8.60	3.57 [1.32, 5.81]	0.002

CIM, change in mean; IKD, isokinetic dynamometer; PADS, portable articulated dynamometry system.

## Discussion

This study evaluated an original dynamometer for the assessment of maximal quadriceps strength in 72 participants. The design of the system was introduced, the agreement with IKD, intra-rater reliability, and interrater reliability were examined, and participants’ experience with the device was investigated. The main results are that the PADS showed excellent correlation with IKD (Pearson’s r: 0.880; *p* < 0.0001) and excellent intra-rater and interrater reliability (Pearson’s r: 0.958 and 0.912; *p* < 0.0001 and < 0.0001, respectively).

In clinical settings, manual muscle testing is the most widely employed means of muscle strength assessments^15,16^; HHDs are also frequently used to quantify muscle strength^18^. MMT and HHDs are popular because they are easy and quick to administer; however, both methods have been reported to demonstrate suboptimal accuracy and reliability^14,17,19^. While IKDs provide precise and accurate measurements, they are not suitable for routine examination due to their size and cost^17,18^. The current study recognized the clinical need to develop a dynamometry system that was portable, easy-to-use, and accessible.

The PADS is a foldable, wearable device that can measure the maximal isometric knee extensor torque in various patient positions. With the weight and size of a typical textbook, the PADS is noticeably smaller and more portable than an IKD. The PADS employs an intuitive design with simple material; the aluminum frame and 3D printed calf/thigh supports are easy to manufacture, and the embedded high precision sensor is easily replaceable. Production of the current prototype only cost about $700 in materials. Good maintainability and cost efficiency are two strengths that the PADS offers, in addition to reliability and portability. Although not yet commercially available, an international patent of the PADS via the Patent Cooperation Treaty (PCT) system is pending, and the production line is in the process of registering a good manufacturing practice (GMP) certificate to make the PADS more readily accessible to researchers, clinicians, and potentially the general public.

The current study results demonstrate that the PADS shows good agreement with IKD. In a study of 56 patients with inclusion body myositis, Hogrel et al*.*^14^ reported IKD’s intra-rater SEM to be 8.02%. Our results found the intra-rater SEM of the IKD to be 5.48%, which is comparable with their findings. The intra-rater SEM of the PADS was 8.38%, competing with the results of Hogrel et al. Although the MDC values of the PADS are slightly higher than those of IKD, they are comparable to the corresponding values of existing literature because the MDC is directly proportional to SEM. The interrater reliability of the PADS and IKD were lower than the corresponding results of the intra-rater measurements: the SEM of the PADS and IKD were 5.51% and 4.20%, respectively. The PADS did not exhibit significant differences in the means for interrater reliability or intra-rater reliability evaluations, and with ICC values close to 1, both the PADS and IKD reported excellent relative reliability.

Knee extensor torque, along with maximal grip strength, is reported to be an important clinical indicator for predicting survival^22^, disease course^23^, and quality of life^24–26^ in many neurological^27,28^, musculoskeletal^29,30^, and geriatric^31^ conditions. In the most recent definition of sarcopenia by the European Working Group on Sarcopenia in Older People, gait performance tests, such as gait speed test, short physical performance battery, timed up and go test, and 400-m walk test, are essential for assessing the severity of sarcopenia^11^. Gait performance was reported to be strongly determined by antigravity muscle strength, of which knee extensors are the most important^32,33^. In a 2008 meta-analysis of nearly two million apparently healthy individuals, Garcia-Hermoso et al*.*^2^ found that quadriceps strength was an independent risk factor of all-cause mortality.

In the user experience analysis, participants responded that they felt the PADS was easier, more efficient, clearer, more inventive, and more innovative than the IKD. Not shown in the results section is a preplanned subgroup analysis of participants who were healthcare professionals. About 40% of the cohort (n = 28) were healthcare professionals (e.g. physicians, nurses, physical therapists, and occupational therapists) working for a university hospital. The subgroup analysis showed that they believed the PADS to be significantly easier to use (Supplementary Table 2).

Interpretation of the study results requires caution due to limitations of the study. As only healthy young adults aged between 20 and 39 years were enrolled, the accuracy and reliability of the PADS are not validated in geriatric populations or individuals with very weak quadriceps strengths. Further studies in older adults and patients with neurological or musculoskeletal disorders are therefore warranted. In such studies, validation studies in more positions (e.g. supine, reclined wheelchair) are necessary. Additionally, the present study only evaluated the measurement of knee extensor strength. In certain conditions (e.g. proximal or distal myopathies), simultaneous monitoring of proximal and distal muscle strengths are required. Currently, the PADS for ankle dorsiflexion and plantarflexion strength measurement is in development. Further research of the PADS on ankle strength assessment is planned for more complete lower extremity strength assessment.

## Conclusion

Precise monitoring of muscle strength is clinically valuable. However, muscle strength assessment usually relies on MMT with limited reliability, or IKDs with limited accessibility. This study found that the PADS offers good reliability, potentially better accessibility, and cost-efficiency in a healthy adult population. The proposed PADS may be an effective clinical alternative to the MMT or IKD.

## Methods

### Participants

**Figure 3:**
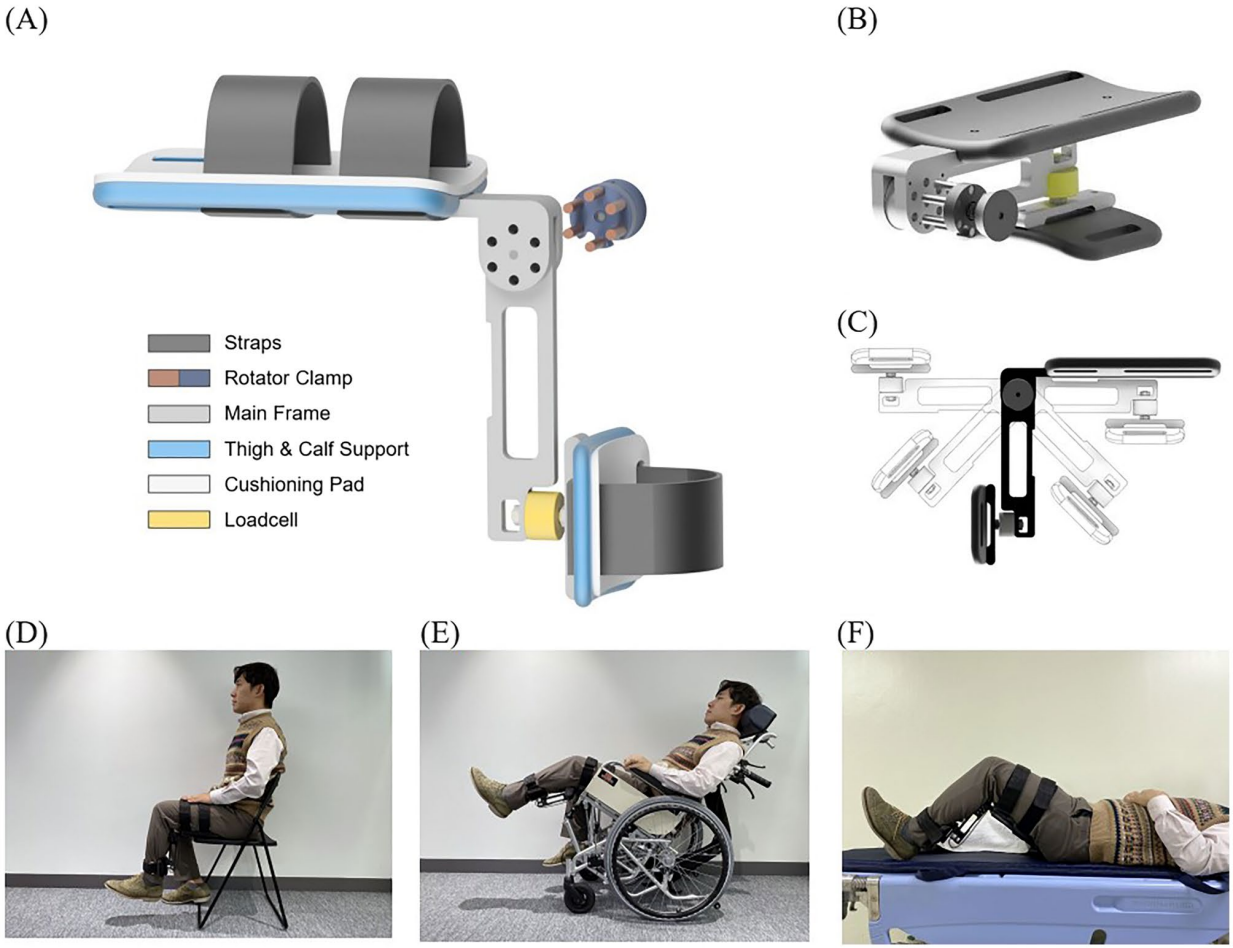
The PADS and its design components (**A**); the rotator-clamp joint of the PADS (**B**) allows fixation at different angles (**C**); subject wearing the PADS in various positions (**D**–**F**).

Seventy-two adult participants between the ages 20 and 39 were enrolled in this study. Participants that were pregnant, had a recent history of cardiovascular, musculoskeletal, neurological, or infectious disease, or could not give informed consent, were excluded. This study conformed to the Declaration of Helsinki. This study was approved by Seoul National University Hospital institutional review board (IRB no. 2201-025-1288). All participants provided written informed consent. The participant appearing in Fig. 3 provided written informed consent to publish the figure in an online open-access publication.

### Anthropometry

A single board-certified physiatrist performed all anthropometric measurements. Measurements were taken with participants barefoot and without heavy clothing. Height was measured to the nearest millimeter with an ultrasonic stadiometer (PUSH Stadiometer; InBody, Seoul, South Korea), and weight was measured to the nearest 0.1 kg using a digital scale (Mi Smart Scale; Xiaomi, Beijing, China). Maximum grip strength was measured with a hand-held grip dynamometer (EH-101; Camry Scale, CA, USA).

### PADS: portable articulated dynamometry system

**Figure 4:**
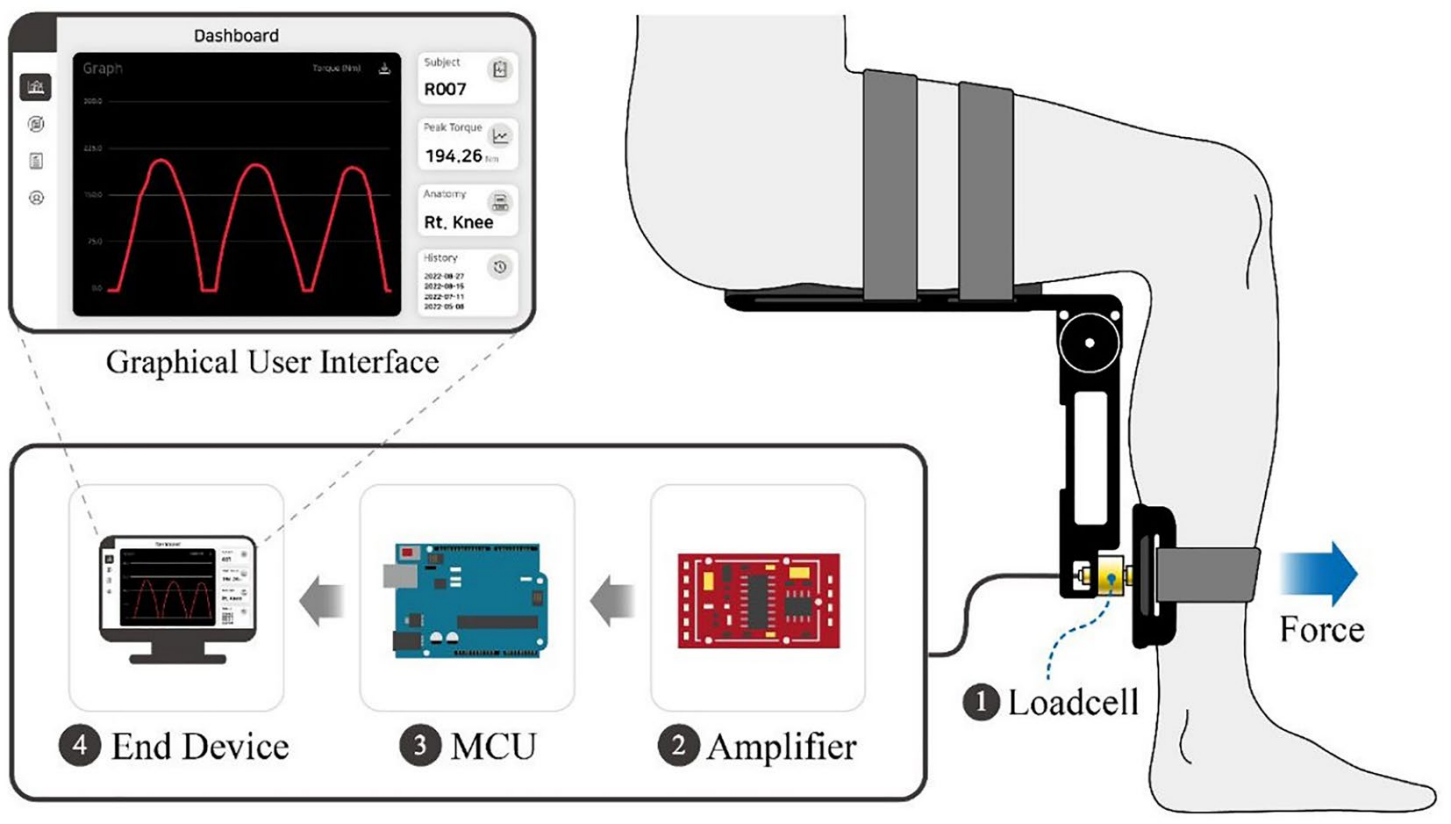
Visualization of the PADS in operation.

The PADS incorporates simple and intuitive mechanical designs to ensure optimal usability and portability (Fig. 3A). The foldable main frame was an aluminum 6061 alloy milled with a Numerical Control milling machine (HF 1250-4; Hyubsung Dynamics Co., Gyeonggi, Korea). The main frame was anodized for insulation, and the joint that that couples the distal and proximal limbs of the frame was a stainless-steel six-pin rotator-clamp that allows the system to either fold completely or peg at a predetermined angle (Fig. 3B). The model used in this study could fold or fixate at thirty-degree increments, allowing maximum portability and isometric torque measurement at different angles (Fig. 3C). Attached to both the distal and proximal limbs of the main frame were ergonomically curved calf and thigh supports. The supports were made of carbon fiber-reinforced nylon filaments additively engineered using a 3D printer (Style Neo A22C; Cubicon, Gyeonggi, Korea), and each support was lined with 10 mm-thick silicon (Ecoflex; Smooth-On, Inc., Macungie, PA, USA) for maximal wearability. The device weighed 1.3 kg, and its dimensions were 250 mm × 120 mm × 100 mm. Embedded between the distal limb of the main frame and the calf support was a high precision push–pull loadcell (CBF30-100; CAS Corporation, Seoul, Korea) that could measure force from 0 to 100 kg, with a maximal hysteresis of 0.5% and repeatability of 0.2% over the entire nominal range. The signal output from the sensor was amplified by an HX711-based amplifier (SEN-13879; SparkFun Electronics, Boulder, CO, USA), and flowed to the microcontroller (Arduino UNO; Arduino, Somerville, MA, USA). The microcontroller delivered the data to the end device, which visualized and recorded the data (Fig. 4). The system was calibrated with a set of class M1 weights from 0.1 to 100 kg; a logarithmic scale calibration curve for the PADS is presented in Supplementary Fig. 1.

To remove the effects of gravity, participants sat on a horizontal Bobath table with their height adjusted so that their lower leg would be perpendicular to, but not rest on, the ground. The proximal limb of the frame was placed under the thigh and fixed with two Velcro belts. The distal limb of the frame, where the tension sensor was embedded, was attached to the calf of the participants with another Velcro belt. Other than encouraging them to remain stable, the examiner did not physically secure the participants in any way after the PADS was attached. The distance between the load cell and lateral epicondyle was measured to the nearest millimeter in twenty participants; the mean distance was used as the lever arm length to compute the torque from the measured force. It was postulated that if the lever arm length estimated from a subset of the population can be used to calculate the knee extensor torque of the entire participant population with acceptable accuracy, thus having been internally validated, then the estimated lever arm length could be used to calibrate the PADS so that subsequent users can use the device without having to measure the lever arm length in every participant. Because the PADS was tightly fastened to their legs, participants were allowed minimal compensatory movements, such as grabbing the edge of the table or slightly leaning forward. Although this study measured maximum isometric knee extension torque at 90° of knee flexion with the participant in a sitting position, the PADS can be worn by participants in the supine position or in wheelchairs, with their knee further extended (Fig. 3D–F).

### Isokinetic dynamometer

Participants sat on an IKD (Biodex System 4 Pro; Biodex Medical Systems, Shirley, NY, USA) with their hip flexed to 85°, and knee joint aligned with the axis of the lever arm of the system. Participants’ hips were stabilized with a safety strap across the pelvis, and the mid-thigh of the tested leg was fastened to the chair with another strap. Participants were seated so that the center of rotation of the lever arm was directly adjacent to the lateral epicondyle.

### Maximal knee extension torque measurement

Three maximal knee extension trials were recorded for each dynamometer; if the last trial showed greater strength than the preceding trials, additional trials were administered until the last measurement was not the greatest record. Before each trial, participants were instructed to execute maximal volition. Strong and continued verbal encouragements were given during the trials, and measurements were performed on both knees; each trial lasted about 3 s, with 30-s breaks in between. To assess the interrater reliability of the two dynamometers, two measurements were performed on each participant by two independent examiners. One examiner was a board-certified physiatrist with 6 years of clinical experience, and the other was an engineer who was part of the development team. Participants were given a 3-min break before switching examiners. To assess the intra-rater reliability of the two dynamometers, a subset of participants (n = 40) went through two repeat assessments, which were at least 24 h apart. All participants performed a 3-min stretching session with a board-certified physiatrist before each assessment, and all assessments were performed under the direct supervision of the same physiatrist.

### User experience study

After completing all assessments, participants were invited to complete the user experience questionnaire short form (UEQ-S) regarding their experience with both dynamometers. The UEQ-S is a reliable and valid tool that allows quick assessment of the feelings, impressions, and attitudes that arise when one uses a product^34^. The questionnaire comprises eight items, and participants rate each item on a 7-point Likert scale, which ranges from − 3 (very negative experience) to + 3 (very positive experience). Completion of the UEQ-S was voluntary, and the responses were anonymous.

### Statistical analysis

The Kolmogorov–Smirnov test was used to assess data normality. CIM and paired *t*-tests were used to detect any systematic bias regarding PADS-IKD cross validation, interrater reliability, and intra-rater reliability. CIM was calculated by taking the mean of differences between the PADS and IKD values of the same participant; CIM was divided by the ratio of standard deviation to the square root of the number of participants to evaluate if there were statistically significant differences in the means between PADS and IKD. To assess reliability and reproducibility, intraclass correlation coefficients were calculated. The agreement between the PADS and the IKD were evaluated using ICC model 2, or tests for absolute agreement. Interrater reliability for each dynamometer was also evaluated using ICC model 2. Intra-rater reliability for each dynamometer was assessed using ICC model 3, or tests for consistency. Because the presented study conducted *m* repeated measures within *n* subjects, the observations were not truly independent. Therefore, ICC_(2,k)_ and ICC_(3,k)_ were used. ICC values less than 0.5, between 0.5 and 0.7, between 0.75 and 0.9, and greater than 0.9 were indicative of poor, moderate, good, and excellent relative reliability, respectively^35–37^. To evaluate absolute reliability, standard error of measurement (SEM) was calculated according to the COnsensus-based Standards of the selection of health Measurement INstruments (COSMIN) quality assessment^38^. Specifically, SEM was calculated using the following formula: $SEM=SD \sqrt{1-ICC}$, where SD represents standard deviation. To provide a more trackable parameter for clinicians and researchers to use as a valuable guide for interpreting changes in the outcomes over time, minimal detectable change (MDC) at 95% confidence level was calculated using the following formula: $MDC=1.96*\sqrt{2}*SEM$. Regression analyses were performed, Bland–Altman plots were created^39^, and the UEQ-S responses for the PADS and IKD were compared using paired *t*-tests. All statistical analyses were performed using SPSS version 26 for Windows (IBM, Armonk, NY, USA). Statistical significance was set at *p* < 0.05.

## Supplementary Information

Supplementary Information.

## Data Availability

The data acquired in this study are not openly available due to the sensitive nature of human data (e.g. age, sex, height and weight). A de-identified dataset containing the full demographic and clinimetric data is available from the corresponding author (S.K.) upon reasonable request.
