# Supplementary material for: Development and validation of a portable articulated dynamometry system to assess knee extensor muscle strength
Source: Sci Rep. 2023 Jul 23;13:11887. doi: 10.1038/s41598-023-39062-0 (PMC10363537; doi:10.1038/s41598-023-39062-0)
Supplement: Supplementary file 1 — Supplementary Information. [file 41598_2023_39062_MOESM1_ESM.docx]

**Supplementary Table 1.** Demographic information of participants

| **Study population** (n=72) | |
| --- | --- |
| **Age** |  |
| 20-29 (n, %) | 36, 50.0% |
| 30-39 (n, %) | 36, 50.0% |
| Mean age (years) | 29.32±4.57 |
| **Sex** |  |
| Male (n, %) | 36, 50.0% |
| Female (n, %) | 36, 50.0% |
| **Height** (cm, mean±std) | 168.48±7.01 |
| **Weight** (kg, mean±std) | 65.31±16.66 |
| **Maximal grip strength** (kg, mean±std) | 37.91±11.99 |

Abbreviation: std, standard deviation.

**Supplementary Table 2.** UEQ-S results of healthcare professionals (n=28)

|  | **IKD** | **PADS** | ***p*-value** |
| --- | --- | --- | --- |
| Supportive | 1.21±1.29 | 1.14±1.33 | 0.81 |
| **Easy** | **0.5±1.77** | **1.64±1.54** | **0.005** |
| Efficient | 1.11±1.43 | 1.40±1.17 | 0.264 |
| Clear | 1.43±1.17 | 1.64±1.16 | 0.433 |
| Exciting | 1.11±1.43 | 1.21±1.47 | 0.699 |
| Interesting | 1.11±1.59 | 1.39±1.4 | 0.341 |
| Inventive | 0.79±1.62 | 1.39±1.31 | 0.088 |
| **Leading edge** | **0.46±1.48** | **1.32±1.33** | **0.011** |
| Total score | 7.71±10.11 | 9.18±9.38 | 0.085 |

Abbreviation: IKD, isokinetic dynamometer; PADS, portable articulated dynamometry system


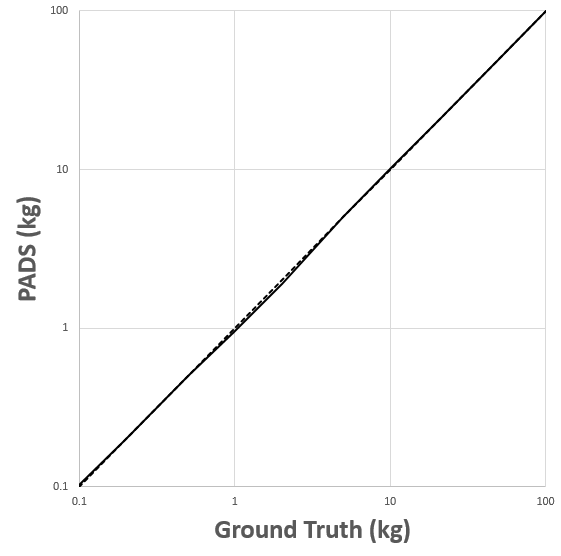


**Supplementary Figure 1.** Calibration Curve of the PADS. Dotted, identity line; Solid, calibration curve.
